# Supplementary material for: Loci for human leukocyte telomere length in the Singaporean Chinese population and trans-ethnic genetic studies
Source: Nat Commun. 2019 Jun 6;10:2491. doi: 10.1038/s41467-019-10443-2 (PMC6554354; doi:10.1038/s41467-019-10443-2)
Supplement: Supplementary file 2 — Supplementary Information [file 41467_2019_10443_MOESM2_ESM.pdf]

Supplementary Information for

Loci for human leukocyte telomere length in the Singaporean Chinese population and trans-ethnic genetic studies.

Dorajoo R et al.

Supplementary table 1: Association of age and sex in the combined SCHS dataset. Age effects adjusted for sex and principle components (PC1-3) and sex effects adjusted for age and principle components (PC1-3). Males coded and 2 and females coded as 1 in regression analyses.

	**Beta**	**SE**	**P**	**% variance explained**
**Age**	-0.033	0.0008	1.03E-126	6.30
**Sex**	-0.227	0.0125	2.57E-51	1.29

Supplementary table 2: Changes to adjusted R^2 values in regression models (adjusted for age, sex and PC1-3) with and without the inclusion of 16 genome-wide associated SNPs. Association analyses performed separately in SCHS Discovery, SCHS replication and combined SCHS samples.

	N	**Adj R^2 in model including 16 SNPs**	**Adj R^2 in model excluding 16 SNPs**	**Difference in R^2**
**SCHS Discovery**	16,759	0.1265	0.0810	0.0455
**SCHS Replication**	6,337	0.1169	0.0831	0.0338
**SCHS combined**	23,096	0.1215	0.0817	0.0398

Supplementary table 3: Evaluation of 16 identified SNPs in additional Singaporean Chinese type 2 diabetic cases. P_{Adj}: Association P adjusted for 16 tests; TA: Test allele; P_{Het}: indicates Cochran's Q P for heterogeneity of effects.

					DN (N=624)				SMART2D (N=978)				Meta-analysis (N=1,602)				
**SNP**	**Chr**	**Position**	**Gene**	**TA**	**TAF**	**Beta**	**SE**	**P**	**TAF**	**Beta**	**SE**	**P**	**Beta**	**SE**	**P**	P_{Adj}	P_{Het}
rs41293836	14	24721327	*TINFS2*	C	0.918	-0.248	0.104	0.017	0.907	-0.142	0.073	0.071	-0.183	0.061	0.003	0.048	0.391
rs7705526	5	1285974	*TERT*	C	0.585	-0.03	0.056	0.599	0.605	-0.098	0.049	0.047	-0.068	0.037	0.066	1	0.359
rs2293607	3	169482335	*TERC*	C	0.539	0.035	0.059	0.548	0.534	-0.05	0.046	0.279	-0.018	0.036	0.628	1	0.255
rs12415148	10	105680586	*OBFC1*	T	0.945	-0.069	0.122	0.572	0.939	-0.028	0.098	0.778	-0.044	0.076	0.566	1	0.793
rs3219104	1	226562621	*PARP1*	A	0.401	-0.026	0.055	0.645	0.456	0.014	0.046	0.604	-0.004	0.035	0.917	1	0.493
rs28365964	8	73920883	*TERF1*	T	0.983	-0.085	0.289	0.681	0.982	-0.16	0.21	0.446	-0.134	0.17	0.43	1	0.834
rs10857352	4	164101482	*NAF1*	A	0.752	-0.155	0.063	0.014	0.736	-0.066	0.052	0.201	-0.102	0.04	0.011	0.176	0.276
rs41309367	20	62309554	*RTEL1*	T	0.277	-0.032	0.064	0.613	0.276	-0.1	0.054	0.063	-0.072	0.041	0.081	1	0.414
rs227080	11	108247888	*ATM*	G	0.417	0.002	0.057	0.969	0.412	-0.031	0.046	0.503	-0.018	0.036	0.619	1	0.651
rs7776744	7	124599749	*POT1*	G	0.574	0.047	0.058	0.416	0.561	0.021	0.048	0.659	0.031	0.037	0.391	1	0.728
rs7253490	19	22293706	*ZNF257*	C	0.671	0.028	0.061	0.642	0.663	0.02	0.05	0.691	0.023	0.038	0.547	1	0.913
rs2967374	16	82209861	*MPHOSPH6*	G	0.849	0.182	0.078	0.02	0.837	-0.011	0.062	0.86	0.064	0.049	0.191	1	0.054
rs11890390	2	54485682	*ACYP2*	C	0.811	0.068	0.068	0.319	0.812	0.07	0.061	0.252	0.069	0.045	0.129	1	0.982
rs2302588	14	73404752	*DCAF4*	G	0.774	-0.077	0.068	0.257	0.787	-0.017	0.057	0.766	-0.041	0.043	0.34	1	0.498
rs7095953	10	101274425	*NKX2-3*	C	0.464	-0.078	0.058	0.18	0.459	-0.015	0.047	0.755	-0.04	0.037	0.277	1	0.399
rs1001761	18	662103	*TYMS*	A	0.701	-0.047	0.062	0.445	0.701	-0.099	0.051	0.052	-0.078	0.039	0.047	0.752	0.514

Supplementary table 4: Association of rs7253490 SNP with mortality due to pneumoniae/influenza and chronic obstructive pulmonary disease (COPD). Associations adjusted for age, sex, PC1-3, BMI and smoking status (never smokers vs current/ex smokers).

				**Pneumoniae/Influenza (1,027 cases / 22,105 controls)**		**COPD (196 cases / 22,936 controls)**	
**SNP**	**Chr**	**Position**	**Test allele**	**HR (95% CI)**	**P**	**HR (95% CI)**	**P**
rs7253490	19	22293706	C	1.173 (1.069 - 1.287)	7.44E-04	1.218 (0.983 - 1.508)	0.071

Supplementary table 5: Mediation analysis summary statistics for total, direct and indirect effects of rs2753490 on respiratory infection deaths. Proportion of total effect mediated through LTL calculated as indirect effect divided by total effect. All analysis adjusted for age, sex and PC1-3.

	**Coefficient**	**SE**	**P**
**Total effect of rs7253490 on respiratory disease deaths**	0.0061	0.0019	0.001
**Direct effect of rs7253490 on respiratory disease deaths**	0.0060	0.0019	0.002
**Indirect effect of rs7253460 on respiratory disease deaths through LTL**	0.0002	0.0001	0.014
**Proportion of total effect mediated through LTL**	3.28%		
**Ratio of indirect to direct effect**	0.025		
**Ratio of total to direct effect**	1.025		

Supplementary table 6: Main clinical characteristics of datasets utilized in the study. Data presented and mean (SD).

**Study**	**SCHS Discovery**	**SCHS Replication**	**SMART2D**	**DN**
N	18,114	7,159	978	624
Ethnicity	Singaporean Chinese	Singaporean Chinese	Singaporean Chinese	Singaporean Chinese
Age	55.1014 (7.4312)	55.0686 (7.4392)	58.6758 (11.5096)	58.2439 (12.2501)
Leukocyte telomere length	1.0203 (0.2239)	1.0119 (0.2168)	0.7095 (0.3051)	0.8514 (0.3193)
% males	44.7524	44.5125	54.7491	60.5712

Supplementary table 7: Interaction analysis of lifestyle factors (smoking status, alcohol consumption and physical activity levels) and BMI with rs7253490 SNP to modify respiratory disease mortality effects. For each interaction analysis, the effects of the interaction term (lifestylexSNP) was adjusted for the specific lifestyle and SNP variables in the same regression model. Smoking: Never smokers coded as 0, former smokers coded as 1 and current smokers coded as 2; Alcohol: never drinkers coded as 0, weekly drinkers coded as 1 and daily drinkers coded as 2; Physical activity: No moderate activity/week coded as 0, 1-3 hours moderate activity/week coded as 1 and >3 hours moderate activity /week coded as 2.

**Interaction term**	**HR**	***P***
Smoking (never/former/current) x rs7253490	1.008 (0.901 - 1.112)	0.892
No. of ciggarettes per day x rs7253490	1.002 (0.994 - 1.010)	0.573
Alcohol (never/weekly/daily) x rs7253490	1.013 (0.819 - 1.252)	0.902
Grams alcohol per day x rs7253490	1.008 (0.996 - 1.020)	0.176
Physical activity x rs7253490	0.974 (0.841 - 1.128)	0.727
BMI x rs7253490	1.016 (0.983 - 1.051)	0.339

Supplementary table 8: Sample QC procedures for the SCHS study samples.

Total samples genotyped	18,114	7,159
Genotyping array	Global Screening Array v1.0	Global Screening Array v2.0
Samples with call-rate < 95%	128	48
Samples with exteremes in heterozygosity (> 3SD or < -3SD)	164	72
Cryptic related samples	1625	
PCA outliers	55	
Samples pass QC	16,823	6,358
Samples with LTL data	16,816	6,355
Samples with extremes in LTL values (> 3SD or < -3SD)	57	18
Remaining samples	16,759	6,337

Supplementary table 9: SNP QC procedures in individual Singaporean Chinese datasets for LTL associations.

	**SCHS Discovery**	**SCHS Replication**	**SMART2D**	**DN**
Total SNPs	22225822	22225822	23872901	22058724
SNPs with call-rate < 95% and rare SNPs (MAF < 1%)	14734972	14734137	15206031	14502252
SNPs with HWE p-value < 10-6	1284	350	27	1
Remaining SNPs that pass QC	7489566	7491335	8666843	7556471
Imputed SNPs with info score < 0.8	1081607	1085097	2144698	767031
SNPs that passed QC	6407959	6406238	6522145	6789440
Genomic inflation factor	1.0426	1.0164	1.0027	0.9942

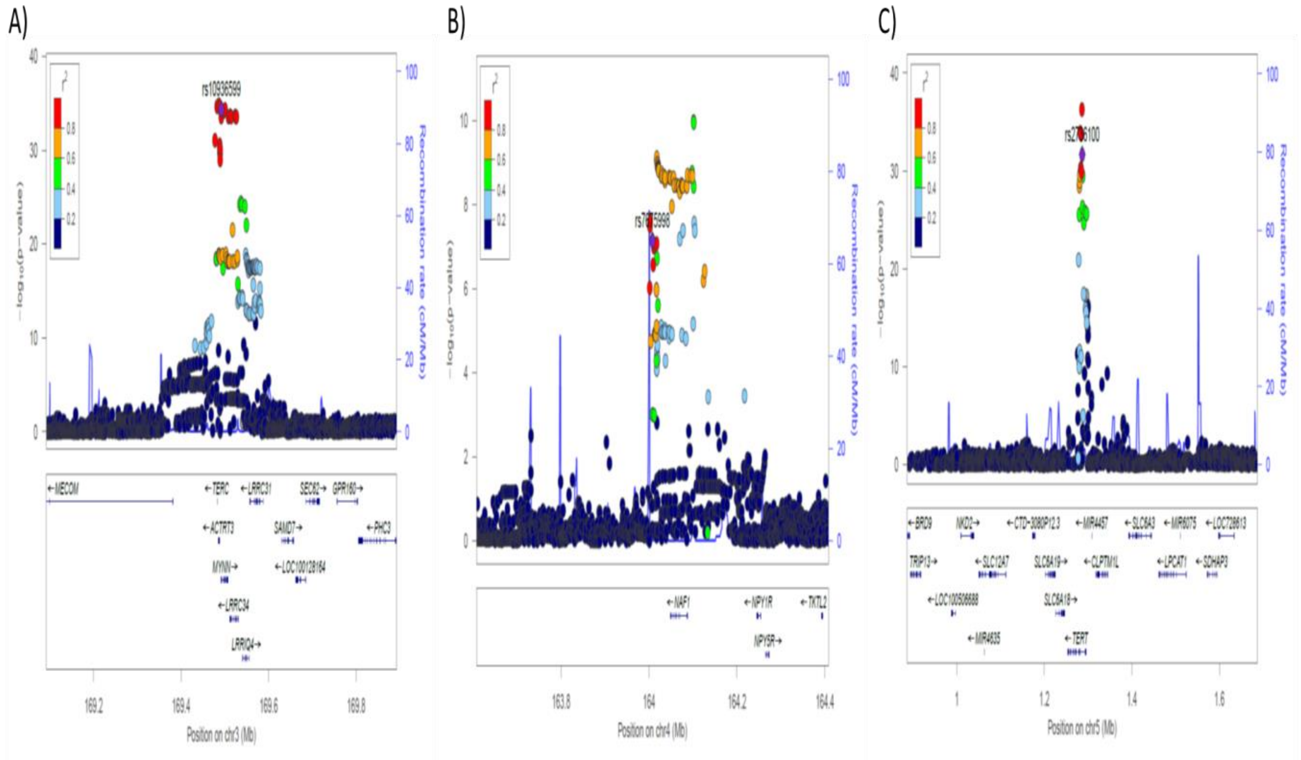

Supplementary Fig 1: Regional plots of genome-wide loci at which index SNPs from previous European GWAS studies were identified to be in LD with the top SNP associations in the SCHS Singaporean Chinese GWAS study. A) Regional association at the TERC gene locus in chromosome 3. B) Regional association at the NAF1 gene locus in chromosome 4. C) Regional association at the TERT gene locus in chromosome 5. Purple diamonds represent previously identified index SNPs. LD (r2) data of SNPs based on ASN panels of 1000Genome database. Plots plotted using LocusZoom (http://csg.sph.umich.edu/locuszoom/).

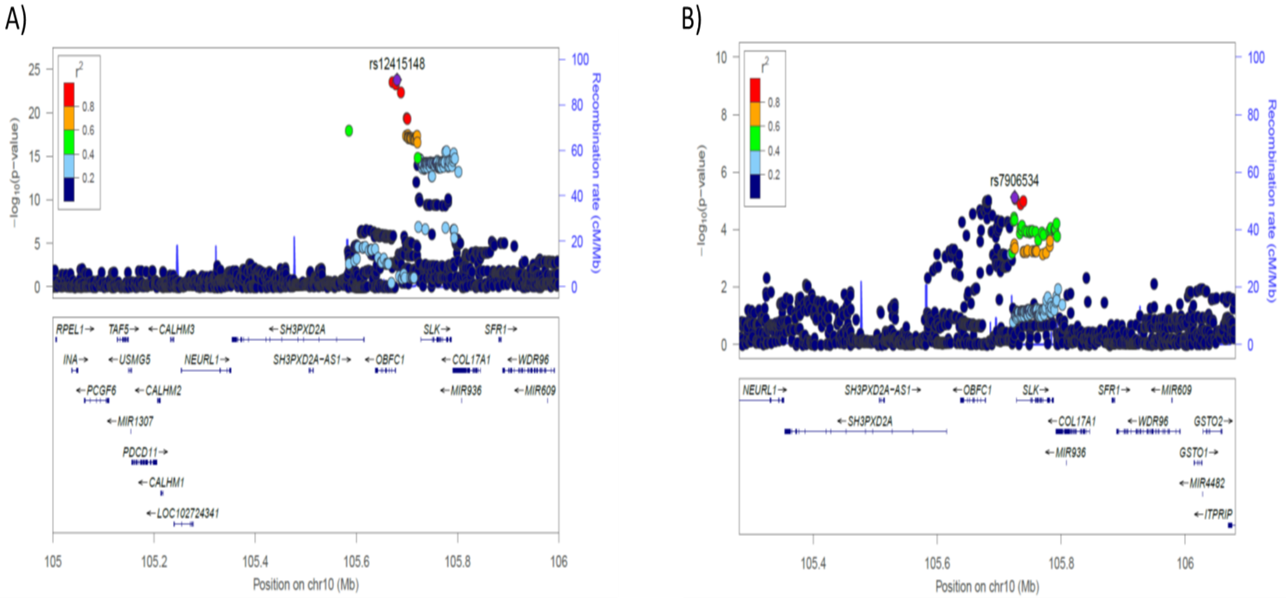

Supplementary Fig 2: Regional plots of SNP associations at the *OBFC1* gene locus in chromosome 10 A) after adjustment on European index SNP genotypes (rs9420907) and B) after adjustment on SCHS lead SNP genotypes (rs12415148). Purple diamond represents top signals after each conditional probability analysis. LD (r^2) data of SNPs based on ASN panels of 1000Genome database. Plots plotted using LocusZoom (http://csg.sph.umich.edu/locuszoom/).

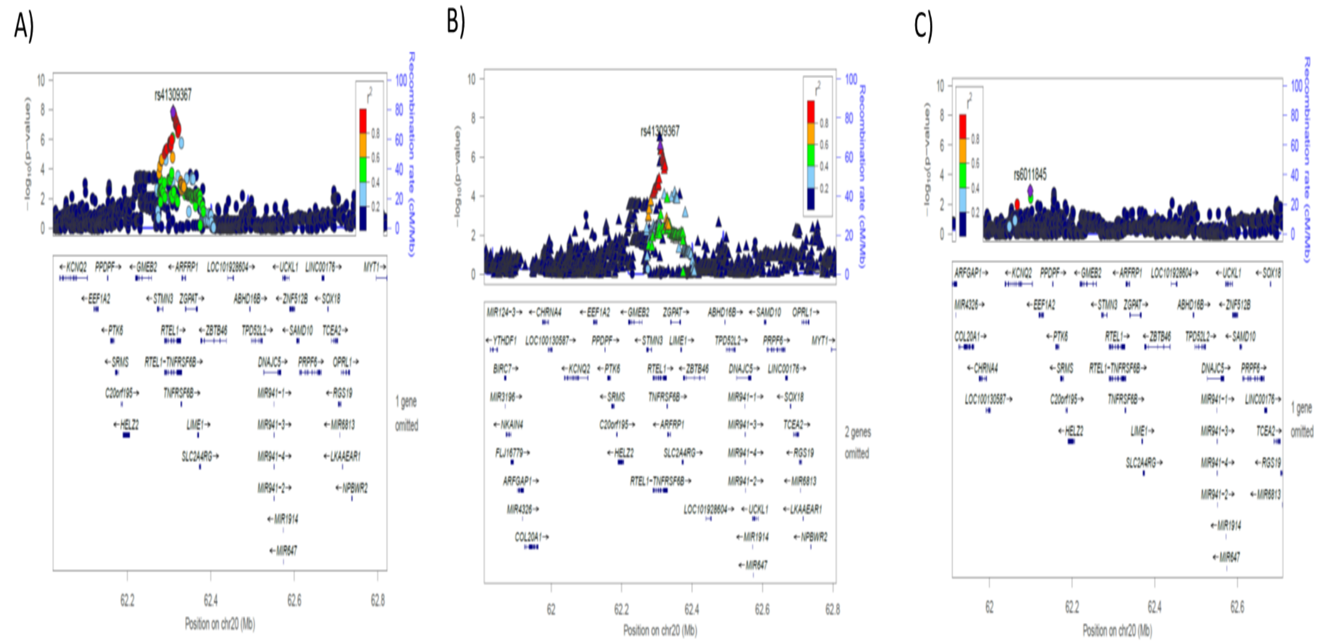

Supplementary Fig 3: Regional plots of SNP associations at the *RTEL1* gene locus in chromosome 20 in the Singaporean Chinese GWAS study A) after adjustment on European index SNP genotypes (rs755017), B) after adjustment on South Asian index SNP genotypes (rs2297439) and C) after adjustment on novel lead SNP genotypes (rs41309367). Purple diamond represents top signals after each conditional probability analysis. LD (r^2) data of SNPs based on ASN panels of 1000Genome database. Plots plotted using LocusZoom (http://csg.sph.umich.edu/locuszoom/).

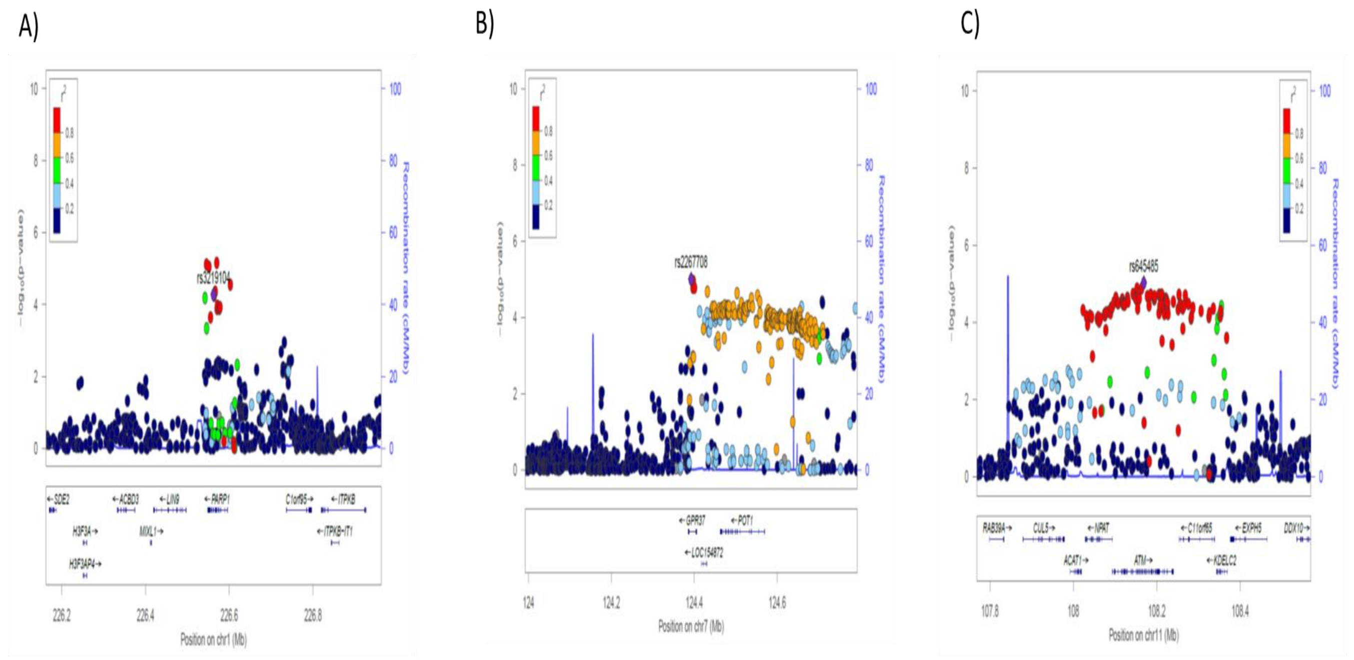

Supplementary Fig 4: Regional plots of LTL SNP associations from ENGAGE European GWAS studies at A) *PARP1* gene locus (rs3219104 was the same lead SNP identified at genome-wide association levels in the SCHS study), B) *POT1* gene locus [(rs2267708 was in LD with SCHS lead SNP, rs7776744, (r^2=0.641 in CEU reference panel)] and C) *ATM1* gene region [(rs645485 was in LD with SCHS lead SNP, rs227080, (r^2=0.940 in CEU reference panel)]. LD (r^2) data of SNPs based on CEU panel of 1000Genome database. Plots plotted using LocusZoom (http://csg.sph.umich.edu/locuszoom/).

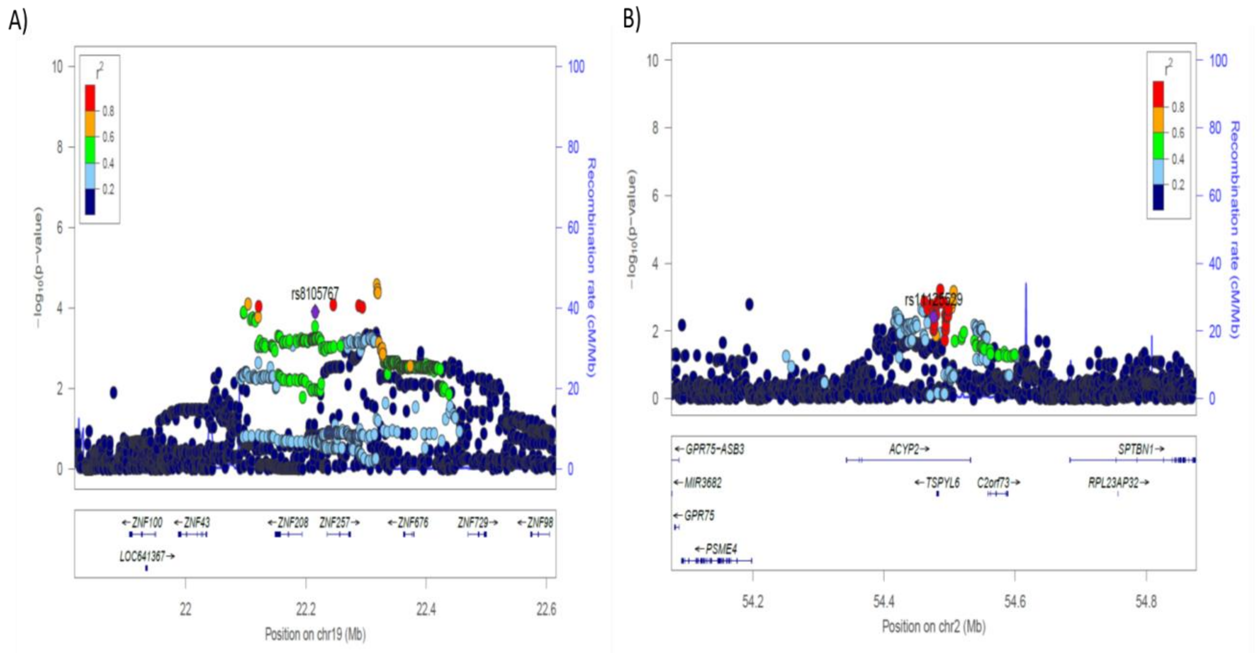

Supplementary Fig 5: Regional plot of SNP associations in the Singaporean GWAS study at A) chromosome 19 locus indicates that the top SNP association in this region (rs7253490) was in LD with the previously identified index SNP (rs8105767) and B) chromosome 2 locus indicates that the top SNP association in this region (rs11890390) was in LD with the previously identified index SNP (rs11125529). Purple diamonds represent previously identified index SNPs. LD (r^2) data of SNPs based on ASN panels of 1000Genome database. Plots plotted using LocusZoom (http://csg.sph.umich.edu/locuszoom/).

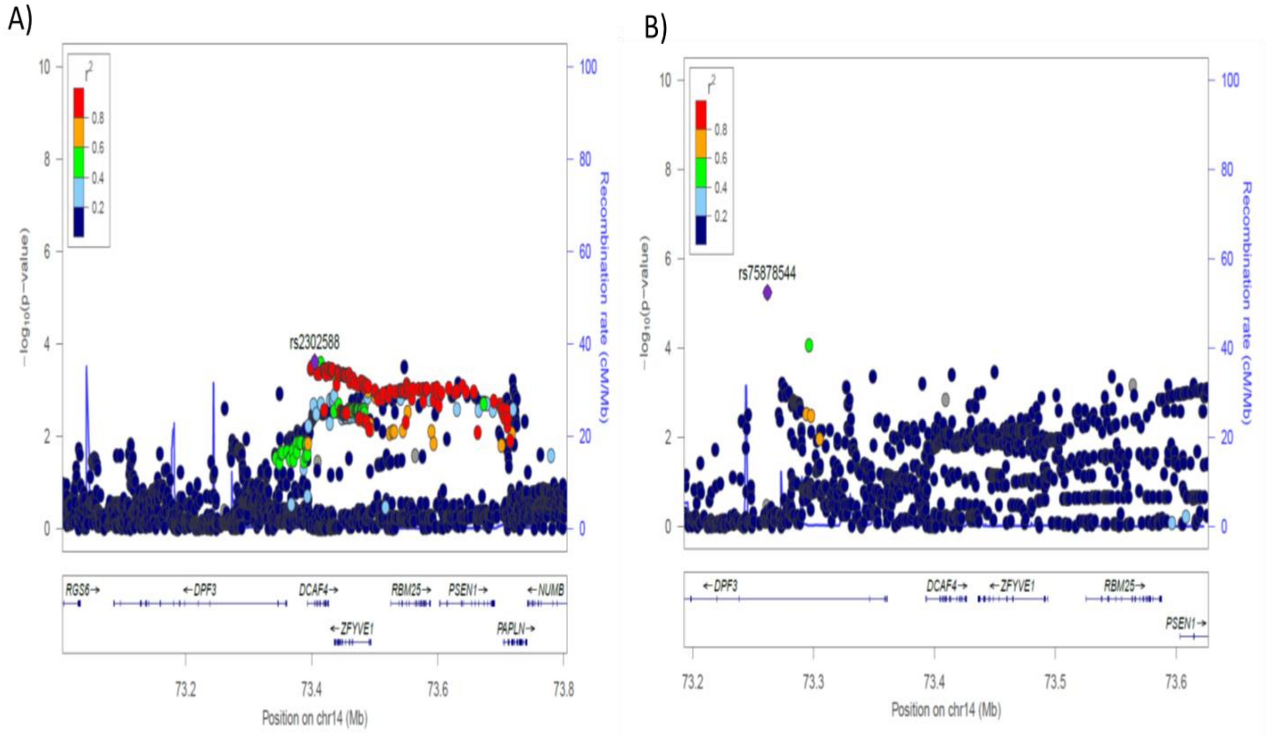

Supplementary Fig 6: Regional plots of SNP associations at the *DACF4* gene locus in chromosome 14 in the Singaporean Chinese GWAS study A) after adjustment on European index SNP genotypes (rs2535913), B) after adjustment on novel lead SNP genotypes (rs2302588). Purple diamond represents top signals after each conditional probability analysis. LD (r^2) data of SNPs based on ASN panels of 1000Genome database. Plots plotted using LocusZoom (http://csg.sph.umich.edu/locuszoom/).

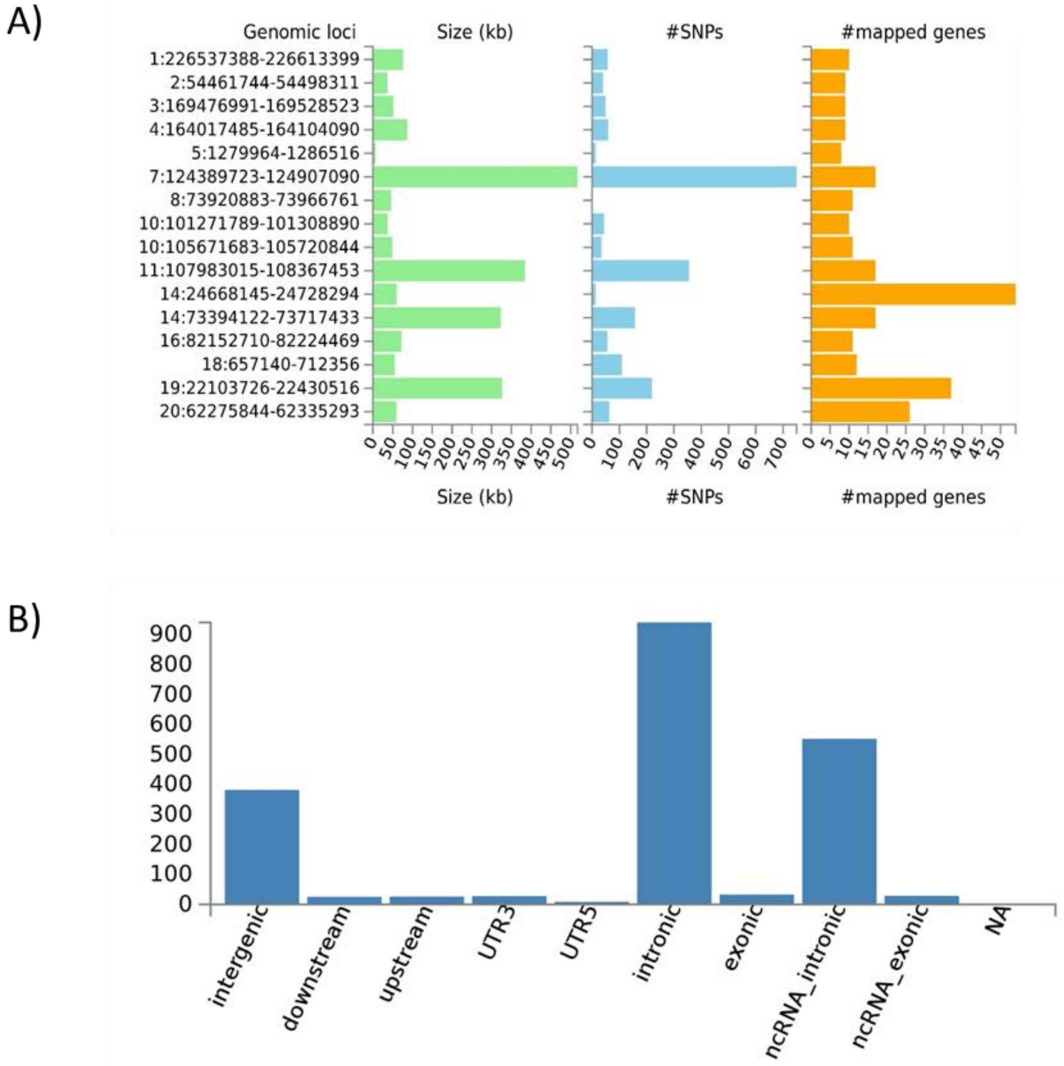

Supplementary Fig 7: FUMA GWAS mapping of SNPs and genes. A) mapping of 2,020 candidate SNPs (r^2 > 0.6 and MAF > 0.1% in 1000G ASN panel) and 268 genes (100kb regional regions from SNP). B) Positional mapping of all 2,020 SNPs in LD (r2 >0.6 1000G ASN panel) with 16 lead SNPs identified in the study.

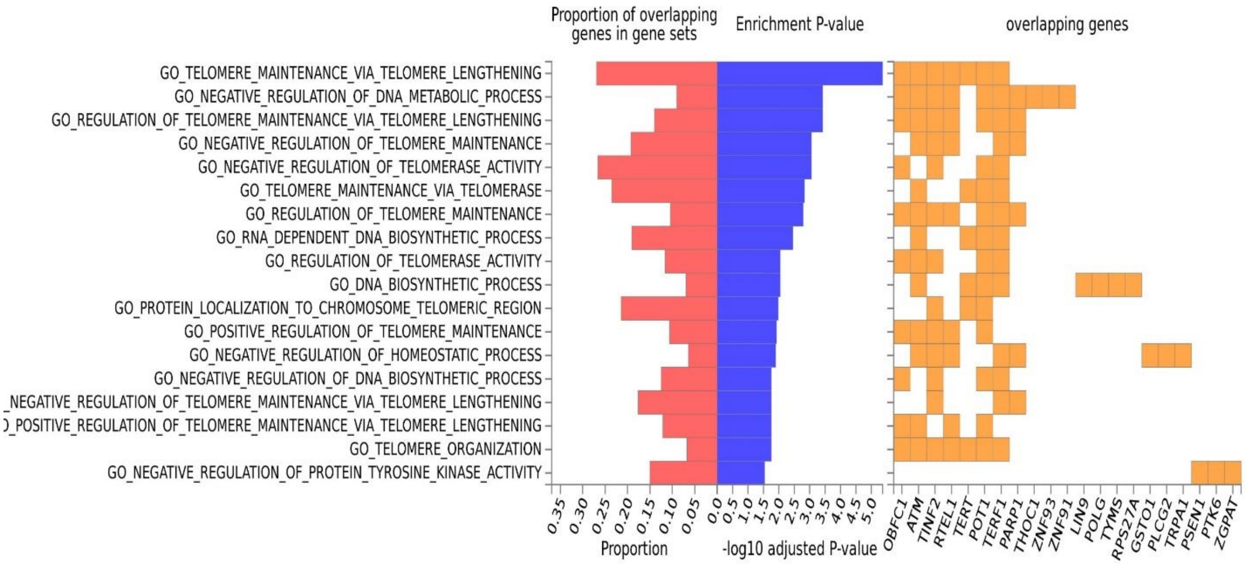

Supplementary Fig 8: Gene Ontology biological process term enrichment for 268 mapped regional genes.

A)

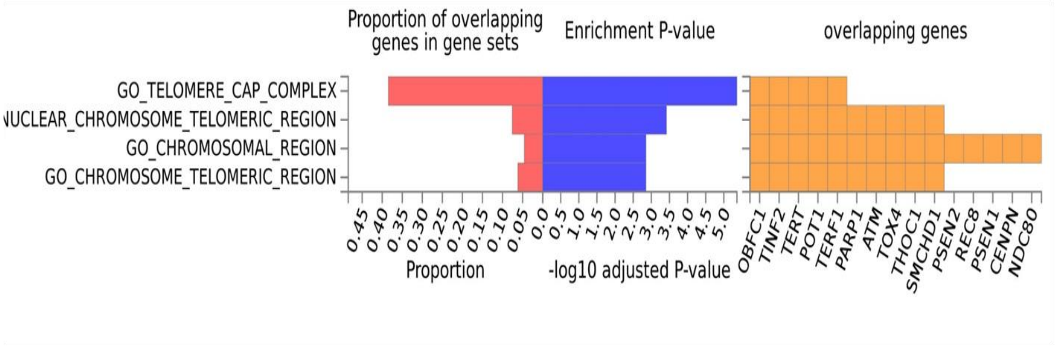

B)

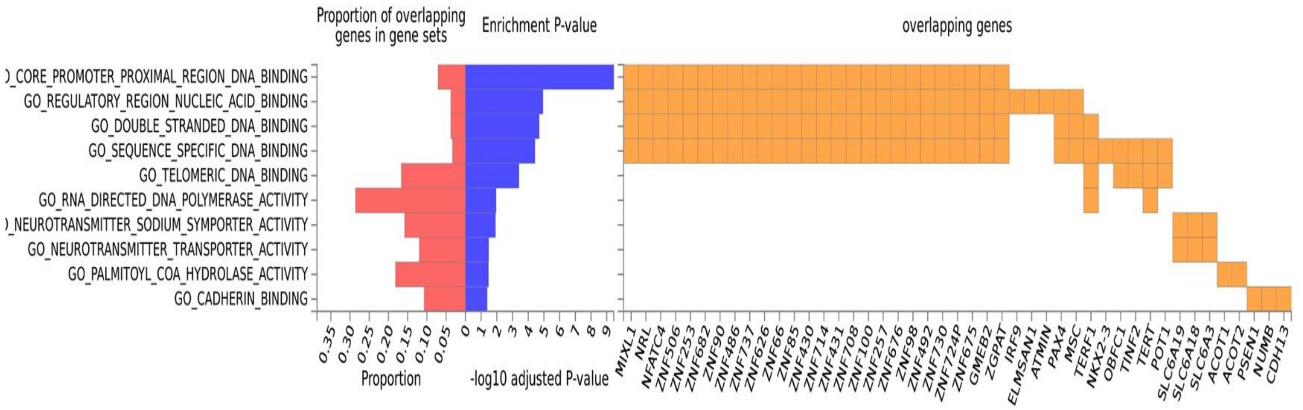

Supplementary Fig 9: Gene Ontology enrichments. A) Gene Ontology cellular component term enrichment and B) gene ontology molecular function term enrichment for 268 mapped regional genes.

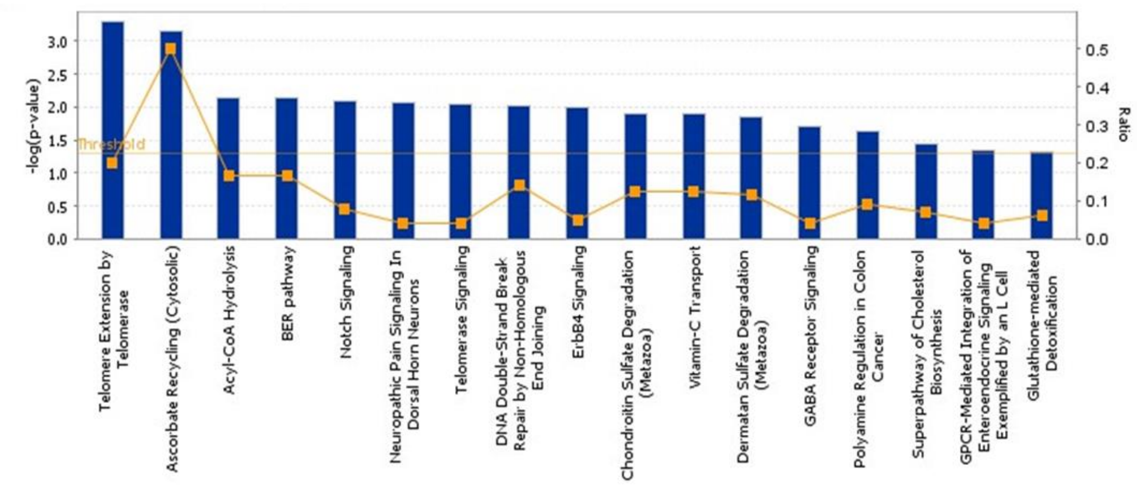

Pathways	Molecules
Telomere Extension by Telomerase	TINF2,POT1,TERF1
Ascorbate Recycling (Cytosolic)	GSTO2,GSTO1
Acyl-CoA Hydrolysis	ACOT2,ACOT1
BER pathway	POLG,PARP1
Notch Signaling	NUMB,PSEN2,PSEN1
Neuropathic Pain Signaling In Dorsal Horn Neurons	KCNQ2,GPR37,GRM8,PLCG2,ATM
Telomerase Signaling	TERT,TINF2,POT1,TERF1,ATM
DNA Double-Strand Break Repair by Non-Homologous End Joining	PARP1,ATM
ErbB4 Signaling	PLCG2,PSEN2,ATM,PSEN1
Chondroitin Sulfate Degradation (Metazoa)	SPAM1,HYAL4
Vitamin-C Transport	GSTO2,GSTO1
Dermatan Sulfate Degradation (Metazoa)	SPAM1,HYAL4
GABA Receptor Signaling	KCNQ2,GPR37,ADCY4,RPS27A
Polyamine Regulation in Colon Cancer	PSME1,PSME2
Superpathway of Cholesterol Biosynthesis	ACAT1,MSMO1
GPCR-Mediated Integration of Enteroendocrine Signaling Exemplified by an L Cell	ADCY4,PLCG2,ADCYAP1
Glutathione-mediated Detoxification	GSTO2,GSTO1

Supplementary Fig 10: Top canonical pathways enriched by 268 mapped genes in the study in Ingenuity Pathway Analysis. Threshold line indicates p-values < 0.05 and ratio indicates percentage of overlapping genes in canonical pathway.

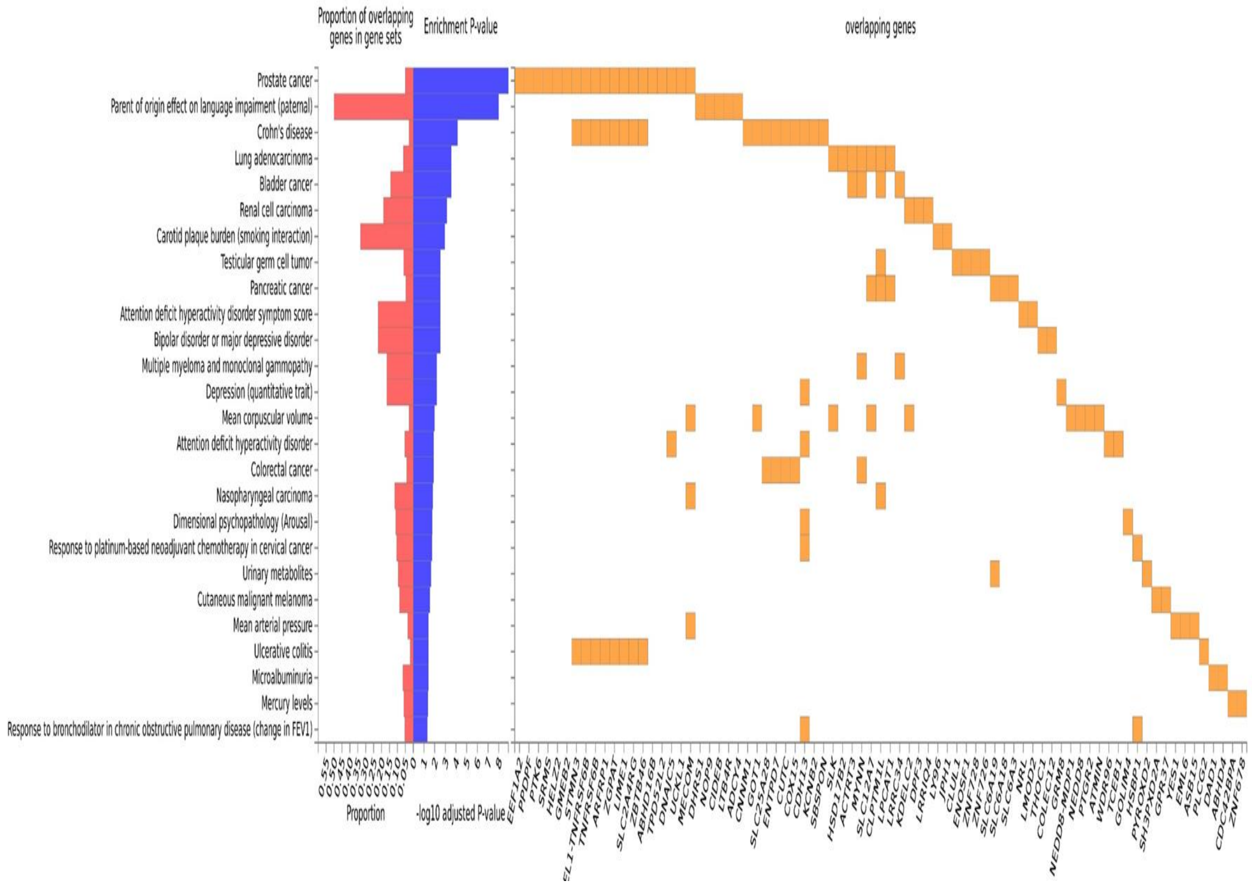

Supplementary Fig 11: Gene set enrichment of mapped regional genes after exclusion of strong telomere-related genes (Table 1) in genetic loci associated with previous GWAS studies. Enrichment P-values adjusted for Bonferroni corrections and proportion indicates percentage of input genes that overlap with genes implicated in previous GWAS traits and diseases.

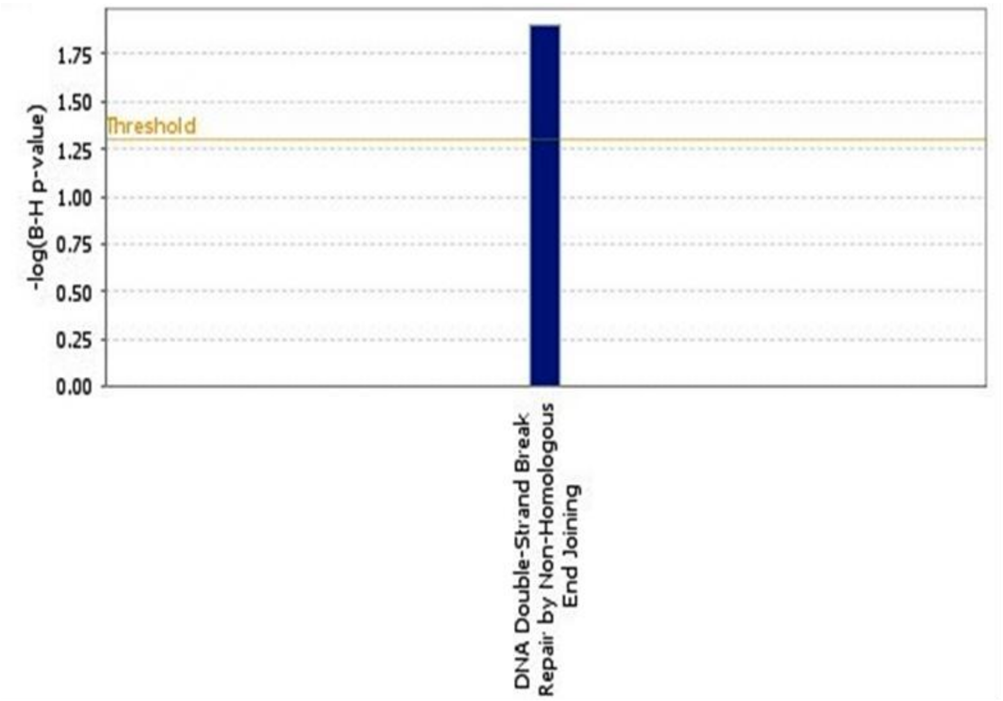

Pathway	Molecules
DNA Double-Strand Break Repair by Non-Homologous End Joining	PARP1,ATM

Supplementary Fig 12: Top canonical pathway enriched by 19 overlapping genes (LTL regional genes with genes for gene expression clock for ageing) in Ingenuity Pathway Analysis. Threshold line indicates Benjamini-Hochberg adjusted $P_{Adj} < 0.05$. The DNA double-strand break repair by non-homologous end joining pathway that included the *ATM* and *PARP1* genes was statistically significant ($P_{Adj} = 0.013$).

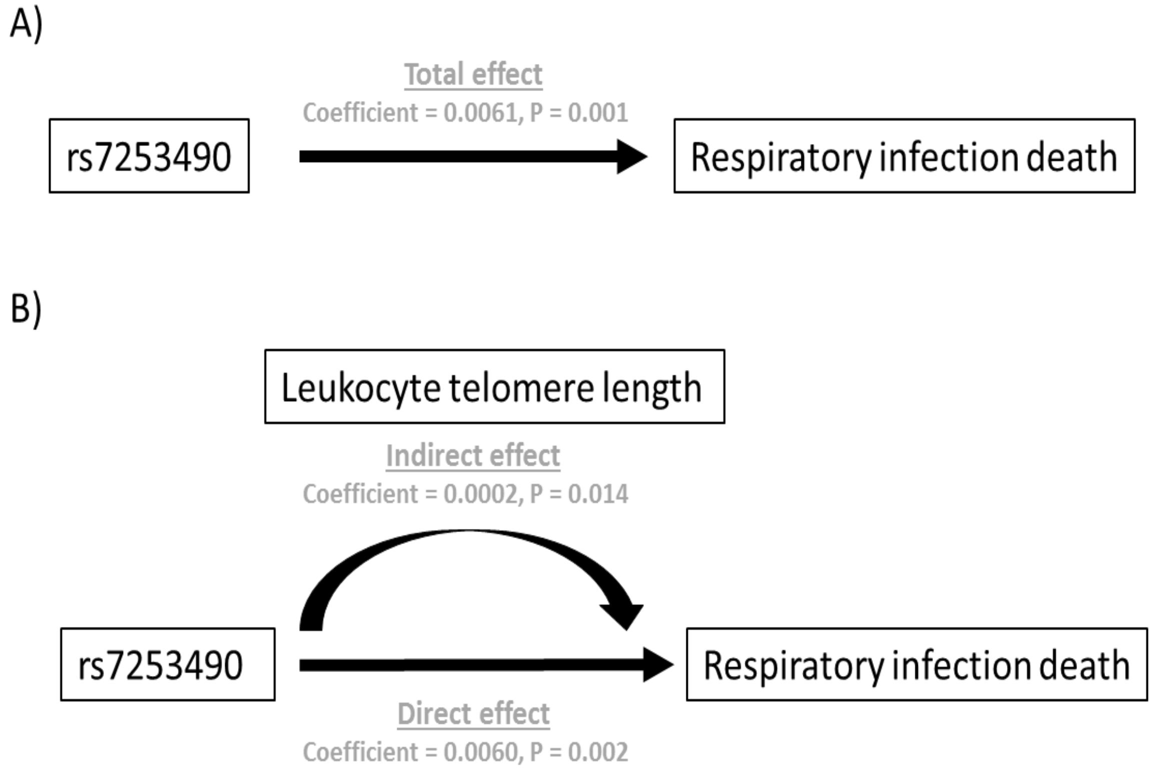

Supplementary Fig 13: Mediation analysis. A) total effect of rs7253490 on respiratory infection death (outcome variable) and B) direct effect of rs2753490 on respiratory infection death through pathways other than leukocyte telomere length (mediating variable) and indirect effect of rs7253490 on respiratory infection death through leukocyte telomere length (mediating variable). All analysis adjusted for age, sex and population substructure (PC1-PC3). Proportion of rs7253490 total effects mediated through leukocyte telomere length = (0.0002/0.0061)x100 = 3.28%.

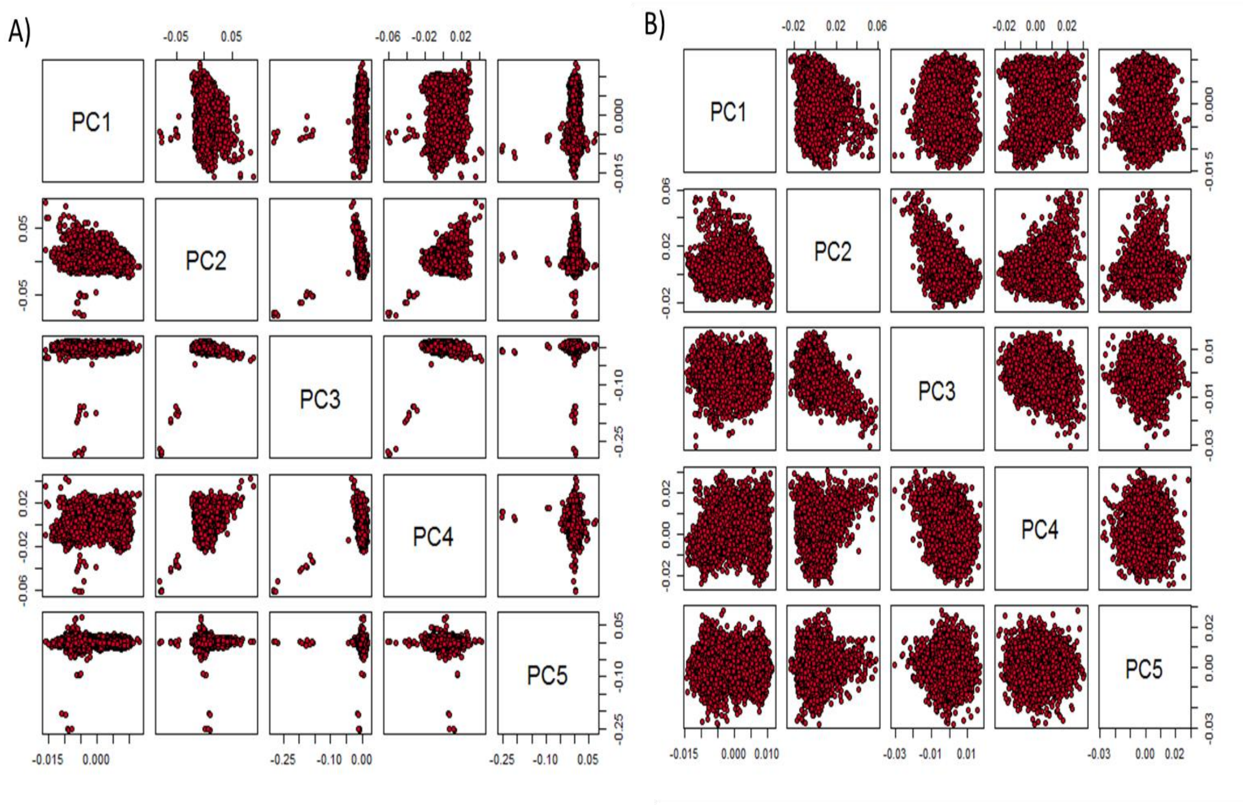

Supplementary Fig 14: PCA analyses (PC1 – PC5) of SCHS study samples. A) before exclusion of PCA outliers and B) after exclusion of 55 PCA outliers.
