# Supplementary material for: Loci for human leukocyte telomere length in the Singaporean Chinese population and trans-ethnic genetic studies
Source: Nat Commun. 2019 Jun 6;10:2491. doi: 10.1038/s41467-019-10443-2 (PMC6554354; doi:10.1038/s41467-019-10443-2)
Supplement: Supplementary file 4 — Description of Additional Supplementary Files [file 41467_2019_10443_MOESM4_ESM.docx]

**Description of Additional Supplementary Files**

**File Name: Supplementary Data 1**

**Description:** Summary data of SNP LTL association in the discovery stage, replication stage and meta-analysis stage for SNPs with meta-analysis P < 0.01. Genomic inflation factor after meta-analysis was negligible (λ = 1.005). TAF: Test allele frequency. P_HET: Cochran's Q p-value.

**File Name: Supplementary Data 2**

**Description:** Summary statistics of combined Singaporean Chinese GWAS, ENGAGE European GWAS and combined meta-analysis data for all SNPs with meta-analysis P < 5x10-8. Order of test allele harmonized during meta-analysis. TAF: Test allele frequency. P_HET: Cochran's Q p-value.

**File Name: Supplementary Data 3**

**Description:** Functional characterization of GWAS lead SNPs. 2,020 candidate SNPs were in LD (r2 > 0.6 1000G ASN panel) with 16 significantly associated SNPs identified in the study.

**File Name: Supplementary Data 4**

**Description:** Cell-type specific significant QTL effects from the BLUEPRINT (BLUEPRINT of Haematopoietic Epigenomes) epigenomics study based on 268 regional LTL genes identified from the GWAS study. Beta and SE indicate effects of QTL SNPs that were in LD with GWAS lead SNPs (r2 > 0.6, 1000G ASN panel). r2 indicates LD between GWAS lead SNP and QTL SNP.

**File Name: Supplementary Data 5**

**Description:** List of significant eQTLs for ATM gene expression in the DICE (database of immune cell expression, expression quantitative trait loci and epigenomics) study.

**File Name: Supplementary Data 6**

**Description:** List of 268 regional LTL genes (200kb from GWAS lead SNPs) and overlaps with gene loci for expression-aging and methylation-aging clocks.
